# An unexpected flush of translucent bile in prepapillary stone impaction and the gallbladder *in* *situ* *(with video)*

**DOI:** 10.1002/ccr3.4310

**Published:** 2021-06-10

**Authors:** Vincent Zimmer

**Affiliations:** ^1^ Department of Medicine Marienhausklinik St. Josef Kohlhof Neunkirchen Germany; ^2^ Department of Medicine II Saarland University Medical Center Saarland University Homburg Germany

**Keywords:** bile, biliary stone disease, endoscopic retrograde cholangiopancreatography, endoscopic ultrasound

## Abstract

‐ translucent, formerly referred to as “white bile,” is devoid of bilirubin and bile acids due to a lacking gallbladder in continuity.

‐ traditionally attributed to malignant obstruction, rarely acute stone impaction may underlie a clear bile aspect.

## Graphical abstract

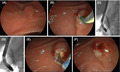

A typical misnomer “white” or rather clear bile is uncommon in benign conditions. This translucent aspect relates to lack of bilirubin and bile acids due to anatomic and/or functional lack of the gallbladder, potentially decompressing the biliary system and concentrating bile fluid.^1^ Thus in an appropriate setting, cystic duct (CD) obstruction can be assumed as illustrated in this 29‐year‐old patient with prepapillary stone impaction. Therefore, we were able to limit radiation exposure (no balloon‐occlusion cholangiogram to confirm CD obstruction) and to limit contrast media exposure with potential risks of infectious complications with clear bile considered to have reduced antimicrobial capacities.^2^ (Figure 1).

**FIGURE 1:**
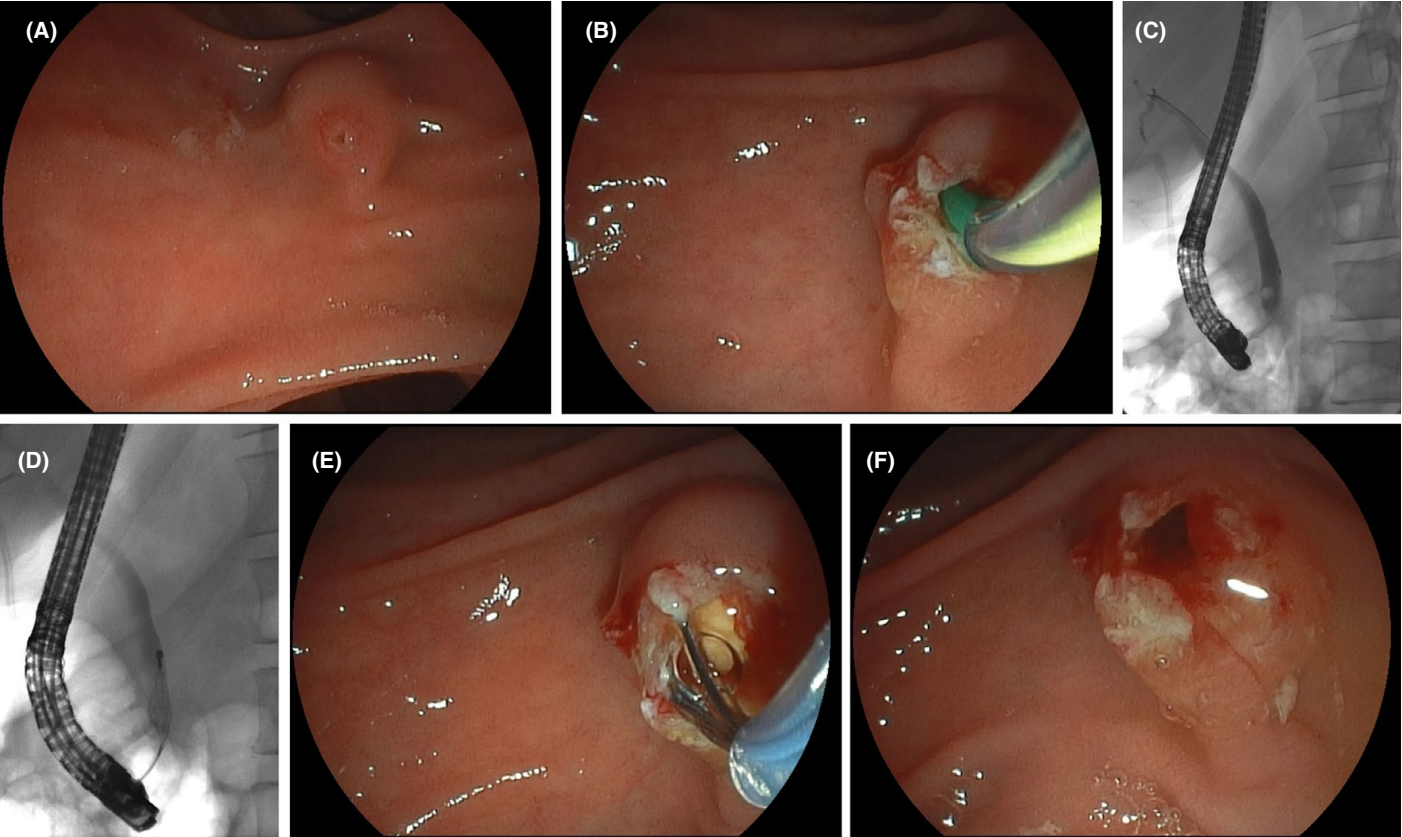
A, Duodenoscopy indicating marked papillary edema compatible with prepapillary stone impaction. B, Successful primary needle knife papillotomy (NKP) access with exuberant drainage of translucent bile (please refer to Video S1). C, A small stone on cholangiogram mobilized upwards during access. D, 8‐wire Dormia basket capture and E, extraction. F, Sufficiently wide NKP incision without need for extensional wire‐guided papillotomy

## CONFLICT OF INTEREST

None declared.

## AUTHOR CONTRIBUTIONS

VZ: involved in clinical care, drafting, and finalization of manuscript.

## ETHICAL APPROVAL

This article does not contain any studies with human participants and/or animals.

## CONSENT STATEMENT

Published with written consent of the patient.

## Supporting information

Video S1

## Data Availability

Data available on request due to privacy/ethical restrictions.
